# 76352 Investigating the Association Between Intracerebral Hemorrhage (ICH) and Long-Term Encephalomalacia and Cortical Atrophy

**DOI:** 10.1017/cts.2021.637

**Published:** 2021-03-31

**Authors:** Arjun Adapa, Siri Sahib Khalsa, Joseph Linzey, Aditya Pandey

**Affiliations:** 1 University of Michigan Medical School; 2 University of Michigan Department of Neurosurgery

## Abstract

ABSTRACT IMPACT: Investigating the relationship between ICH volume at presentation and cortical atrophy over time may help to explain the phenomenon of cognitive impairment after ICH. OBJECTIVES/GOALS: Non-traumatic intracerebral hemorrhage (ICH) is the most common type of hemorrhagic stroke. ICH causes both mechanical as well as oxidative injury leading to neurologic deterioration. However, the relationship between ICH volume and brain atrophy is not fully understood. To that end, we aimed to investigate this relationship. METHODS/STUDY POPULATION: A retrospective cohort of adult ICH patients over a 10-year study period were studied. Demographic data, ICH cause, location, laterality, and treatment, as well as other data, were collected. DICOM files of CT or MRI at presentation and all subsequent follow-up imaging up to 5-years post-ICH were obtained. Using a novel semi-automated image segmentation tool developed in MATLAB by our group, ICH volumes were segmented. Using T1w MRI data, ipsi- and contralateral brain volumes were segmented using NeuroQuant, a fully automated software for MRI volumetric processing, for both initial and follow-up MRIs. Ipsilateral and global cerebral volume changes over time were calculated using initial and follow-up MRI images. Spearman rank correlation between volume changes and ICH volumes were calculated for each patient. RESULTS/ANTICIPATED RESULTS: 75 spontaneous ICH patients with adequate imaging follow-up and both early and follow-up MRI comparisons were considered. Of these, 14 patients had MRIs adequate for segmentation by NeuroQuant. There was a positive correlation (R^2 = 0.72, p=0.03) between ABC/2, the traditional ICH volume measuring approach, and our semi-automatic segmentation tool, with ABC/2 tending to overestimate ICH volume. There was an average of -6.51% of total brain volume loss with respect to initial brain volume at follow-up. There was a negative correlation between ICH volume and both global (Spearman rank correlation coefficient (R)=-0.714, p=0.004)) and local atrophy (R=-0.785, p=0.0009), meaning that as ICH volume increases, there is greater brain volume loss. DISCUSSION/SIGNIFICANCE OF FINDINGS: Greater ICH volume is associated with greater brain volume loss both ipsilaterally, reflected as encephalomalacia, and globally. These findings are important as encephalomalacia can result in focal neurologic deficit and other neurological symptoms over time, while global brain atrophy is associated with dementia and cognitive decline.

